# Non-Dominant Genotypes (GII, GIV and GV) of Japanese Encephalitis Virus Exhibit an Elevated Evolutionary Rate in Nature

**DOI:** 10.3390/microorganisms13122792

**Published:** 2025-12-08

**Authors:** Zhijie Wang, Limin Zhen, Kaiyue Wei, Baoqiu Cui, Zeyu Wang, Anum Farid, Xinyue Xia, Xiaofeng Sun, Hong Liu, Guodong Liang

**Affiliations:** 1 Center for Organoid and Genome Research, School of Life Sciences and Medicine, Shandong University of Technology, Zibo 255049, China; wangzhijie0607@hotmail.com (Z.W.); wky24410011008@outlook.com (K.W.); cuibaoqiu1314@hotmail.com (B.C.); wangzeyu0701@hotmail.com (Z.W.); anum.malik911@gmail.com (A.F.); xxydy@hotmail.com (X.X.); sunxiaofeng12345@outlook.com (X.S.); 2 National Key Laboratory of Intelligent Tracking and Forecasting for Infectious Diseases, National Institute for Viral Disease Control and Prevention, Chinese Center for Disease Control and Prevention, Beijing 102206, China

**Keywords:** Japanese encephalitis virus, Japanese encephalitis, phylogenetic analyses

## Abstract

Numerous studies have demonstrated that the Japanese encephalitis virus (JEV) is classified into five genotypes. Historically, JEV GIII and GI were the dominant strains before and after the 1990s, respectively. Recently, the non-dominant genotypes have been implicated in numerous JE outbreaks, posing significant public health challenges. This study conducted a comprehensive phylogenetic analysis of 126 JEVs covering five genotypes from 1938 to 2025 globally. Notably, it is the first study to conduct a time to most recent common ancestor (tMRCA) analysis of JEV GII. The findings indicate the common ancestor of JEV emerged approximately 1081 years ago, followed by the sequential evolution of GV, GIII, GI, GII, and GIV, which are estimated to have originated approximately 237, 184, 138, 130, and 113 years ago, respectively. The overall evolutionary rate of JEV was 3.3 × 10^−4^ substitutions/site/year, with genotype specific rates as follows: GI at 7.3 × 10^−4^, GII at 8.2 × 10^−4^, GIII at 4.4 × 10^−5^, GIV at 1.7 × 10^−3^, and GV at 2.2 × 10^−3^. Significant differences were found between the E protein of recent human-derived JEV strains across five genotypes and the vaccine strain. This aligns with evidence of the current JE vaccine’s reduced efficacy against new strains, underscoring the urgent need for broadly protective JE vaccines.

## 1. Introduction

**Figure 1:**
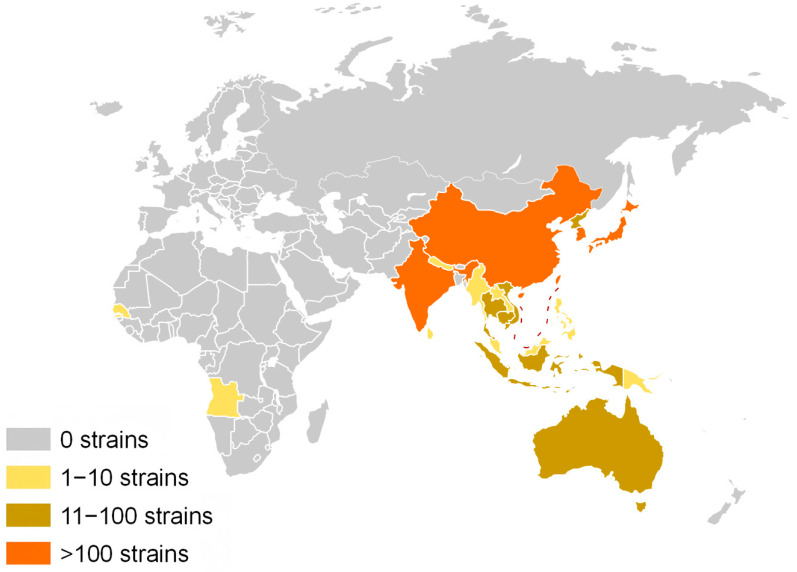
The global distribution of JEV endemic regions is depicted with varying background colors to indicate the prevalence of JEV strains. Regions with no reported distribution of JEV strains are highlighted with a gray background. Areas with a distribution of 1–10 JEV strains are represented with a yellow background, whereas regions with 11–100 strains are marked with a brown background. Finally, areas exhibiting a distribution of over 100 JEV strains are denoted with an orange background. The red dashed line represents the South China Sea maritime boundary.

Japanese encephalitis (JE) is an acute neurotropic infectious disease with a case fatality rate of up to 30%. Among survivors, approximately 45% suffer from long-term neurological sequelae [1]. The disease was first identified in Japan in 1924 and has subsequently been reported in most Asian countries [2]. According to the 2024 epidemiological data from the World Health Organization (WHO), JE is endemic in 24 countries and regions across Asia and the Western Pacific (Figure 1). With an estimated 100,000 cases reported annually, it poses a significant threat to the health and safety of approximately 3 billion people. JE is regarded as one of the most critical viral encephalitides globally [3].

The Japanese encephalitis virus (JEV), the causative agent of JE, is recognized as the prototype virus within the Japanese encephalitis serogroup of the genus Flavivirus, family Flaviviridae. It is characterized as a single-stranded, positive-sense RNA virus with a genome approximately 11 kilobases in length [4]. The JEV genome comprises a single open reading frame (ORF) that encodes three structural proteins (C, prM, and E) and seven non-structural proteins (NS1, NS2A, NS2B, NS3, NS4A, NS4B, and NS5) [5]. The 5′ and 3′ untranslated regions (UTRs) of the genome contain multiple RNA cis-acting elements that form higher-order structures, which play critical roles in viral replication, immune evasion, and the regulation of pathogenesis [6].

Based on the nucleotide sequences of the prM, E, 3′UTR genes and the whole genome, JEV can be classified into five genotypes (GI–GV) [7,8,9,10]. It is widely acknowledged that JEV originated in the India–Malaysia region [8,11], where all five genotypes have been identified, before disseminating across the Asian continent [12]. Historically, GIII was the predominant genotype widely distributed across JEV-endemic regions for decades [13]. Since the 1990s, however, GI has gradually replaced GIII to become the dominant circulating genotype in most parts of Asia [14]. In contrast, GII, GIV, and GV have more restricted geographic distributions and are considered to be “non-dominant” genotypes. GII strains were isolated between 1951 and 1999 from South Korea, southern Thailand, Malaysia, Indonesia, Papua New Guinea, and northern Australia. In 1995, GII caused an outbreak on Badu Island in the Torres Strait, northern Australia, resulting in three human cases, including two fatalities [15,16]. GIV was first identified in Indonesia in the late 1970s, and after nearly half a century of silence, re-emerged in southeastern Australia, where it caused a major JE outbreak that led to 45 human cases and 7 deaths [17,18]. GV was first detected in Malaysia in 1952 and was not reported again until 57 years later, when it was identified in Culex tritaeniorhynchus mosquitoes in China in 2009 [18,19,20]. Since 2010, 21 GV strains have been isolated from adult encephalitis patients and mosquito samples in South Korea, suggesting that GV may have become the predominant genotype in this region [20,21].

It is noteworthy that traditionally considered “non-dominant” JEV genotypes, especially GIV and GV, have re-emerged in nature in recent years and have been closely associated with human encephalitis cases. This observation indicates that JEV is acquiring novel ecological and epidemiological traits throughout its evolutionary process. Nonetheless, our comprehension of the contemporary evolutionary dynamics of JEV in nature, especially concerning the origins and population dynamics of its different genotypes, remains limited.

Although previous studies have provided preliminary insights into the origin and evolutionary trends of JEV, for example, analyses based on 100 complete genome sequences suggested that JEV originated approximately 3255 years ago, with an evolutionary order of GV → GIV → GIII → GII → GI [11]. However, the origin time of GII remains unclear due to the limited availability of genomic data, as only one complete genome sequence of GII was included in earlier analyses. This data bias may have led to inaccuracies in the estimation of JEV’s overall evolutionary history.

To systematically elucidate the evolutionary timescales and population dynamics of JEV genotypes, we assembled a comprehensive global dataset comprising 126 complete genomic sequences, ranging from 1935 to the present. This dataset encompasses all known genotypes, including newly sequenced GII strains. Utilizing this extensive dataset, we conducted a thorough evolutionary analysis to reconstruct the divergence timeline and demographic history of each genotype. Our findings suggest that the sequential emergence of the five JEV genotypes occurred in the order of GV, GIII, GI, GII, and GIV. This proposed timeline challenges previous studies and provides a novel evolutionary framework, thereby offering a scientific basis for understanding viral evolution and anticipating future transmission risks. The detailed findings are presented below.

## 2. Materials and Methods

### 2.1. Dataset Construction

To investigate the phylogenetic relationships among JEV strains, the origin of natural populations, and their recent evolutionary dynamics, all available complete genome sequences of JEV including 67 strains sequenced in our lab were retrieved from the GenBank database as of June 2025. During the data filtering process, strains were excluded if they lacked essential background information, such as the year of isolation, country, or host, or if they had incomplete genome sequences. Additionally, redundant sequencing data, derivative strains, and vaccine strains were removed. Sequence alignment was conducted using MAFFT software v7.526 (https://mafft.cbrc.jp/alignment/software, accessed on 3 February 2025), and sequences exhibiting more than 98% similarity were excluded using R software v 4.43 to ensure the dataset’s diversity and representativeness.

### 2.2. Time-Scaled Phylogenetic Analysis of JEV Based on Complete Genomes

A thorough phylogenetic analysis was undertaken utilizing the complete genome sequences of 126 JEV strains to explore the evolutionary characteristics and natural origins of JEV populations. Recombination detection, conducted using SplitsTree CE v6.1.1.0, indicated no statistically significant evidence of recombination within the dataset (*p* = 0.3053) [22]. Assessment of the phylogenetic signal, performed with IQ-TREE as implemented in PhyloSuite, demonstrated star-like values (*n*) ranging from 2.5% to 30%, suggesting that the dataset contained an adequate phylogenetic signal to support robust evolutionary inference.

Moreover, the analysis of nucleotide substitution saturation utilizing Xia’s test yielded results indicating ISS < ISS.C with a non-significant difference, implying that nucleotide substitutions had not reached saturation. Collectively, these findings confirmed the suitability of the assembled JEV dataset for phylogenetic reconstruction. Model selection conducted via ModelFinder in PhyloSuite v1.2.2 identified GTR + G4 as the best-fitting nucleotide substitution model [23,24]. A Bayesian Markov Chain Monte Carlo (MCMC) analysis was subsequently performed using BEAST v1.10.4 [25] to construct a maximum clade credibility (MCC) tree [26]. Three independent MCMC runs were conducted, each with a chain length of 5 × 10^8^ generations, for reproducibility and convergence validation and to ensure adequate mixing. Based on Bayes factor (BF) comparisons and 95% highest posterior density (HPD) intervals, a Bayesian skyline model with an uncorrelated lognormal relaxed molecular clock was determined to be the best-fitting model [27], and the optimal model was further validated using Bayes factor tests. Parameter convergence and evolutionary rate estimation were evaluated utilizing Tracer software v1.10.4, with a criterion that the Effective Sample Size (ESS) values must meet or exceed 200 to be considered acceptable. The MCC tree was annotated with TreeAnnotator v1.10.4 after discarding the initial 10% of samples as burn-in. Population dynamic history was inferred through Bayesian skyline reconstruction, and the Bayesian MCMC approach was applied to estimate each parameter with 95% HPD intervals representing uncertainty. Posterior probability values were used to evaluate node support across the phylogenetic tree.

To enhance the robustness of the phylogenetic analysis, a maximum likelihood (ML) phylogenetic tree was constructed using IQ-TREE software v2.4.0. The best-fitting nucleotide substitution model, GTR + G4, was selected using ModelFinder in PhyloSuite. Following model selection, the ML tree was inferred, and node support was assessed using the Ultrafast Bootstrap method with 1000 replicates to ensure the reliability of the analysis results.

### 2.3. Structural Similarity Comparison of Genomic Regions Across Five Genotypes of JEV Representative Strains

Multiple sequence alignment of JEV genome sequences was performed using MAFFT v7.526 [28]. Based on genomic structural features, the complete genome dataset was divided into 12 functional regions for analysis using BioEdit v7.0.5.3 [29], including the 5′UTR, the ORF coding regions (C, prM, E, NS1, NS2A, NS2B, NS3, NS4A, NS4B, NS5 genes), and the 3′UTR. Regarding vaccine strain selection, given that the SA14-14-2 strain shares 98.4% genome similarity with the P3 strain (both currently used vaccine strains), P3 was chosen as the reference sequence in this study. Sequence similarity between each genotype strain and the P3 strain was calculated using the MegAlign v7.1.0.44 module in DNAStar (https://www.dnastar.com/, accessed on 8 April 2025) [30,31], and the results were visualized with TBtools v2.376 [32].

### 2.4. Codon Usage Patterns in the Complete Genomes of Different JEV Genotypes

The nucleotide base composition of 126 JEV genome sequences was analyzed utilizing MEGA 11 software [33]. The relative frequencies of adenine (A), thymine (T), guanine (G), and cytosine (C) were determined for each JEV genotype. To illustrate the distribution of base composition across the five JEV genotypes, scatter plots were constructed using the ggplot2 package within the RStudio v2023.12.1+402 environment [34].

### 2.5. Analysis of Amino Acid Mutation Sites in the E Protein of Recent Human JEV Strains Compared to the Vaccine P3 Strain

The latest human-derived JEV strains—GI TWN/2022-EV-H0004/2022, GII FU, GIII JEV1805M, GIV JEV/Human/NT_Tiwi Islands/2021, and GV NCCP 43279—were analyzed in comparison to the vaccine strain P3, focusing on the amino acid sequences and structural characteristics of the E protein. GeneDoc software v2.6.002 facilitated the visualization of sequence mutation sites, enabling the identification of amino acid variations between representative strains of each genotype and the P3 strain.

### 2.6. Structural and Surface Charge Analysis of the E Protein of Recent Human JEV Strains and the Vaccine P3 Strain

Homology modeling of the E protein amino acid sequences was performed using YASARA software v23.9.29, with PDB ID: 7KVA.2.T as the template, to generate monomeric protein structures for the representative strains of the five genotypes and the P3 strain. Structural and surface electrostatic analyses were further conducted using PyMol v3.1.4 and YASARA v23.9.29 [35,36], allowing comparison of the amino acid mutation sites among the different genotype representatives relative to the P3 strain in the three-dimensional structures.

## 3. Results

### 3.1. Dataset Construction

This study incorporated a total of 126 complete JEV genome sequences, encompassing five genotypes: GI (54 strains), GII (8 strains), GIII (47 strains), GIV (9 strains), and GV (8 strains). The temporal span of these strains ranges from 1935 to 2023, with a geographical distribution that includes all regions where JEV has been documented, extending from 15° S to 45° N. Consequently, this dataset offers comprehensive temporal and geographical representation. The host and vector sources predominantly comprised mosquitoes (55 strains), humans (34 strains), pigs (32 strains), and additionally included cattle (1 strain), horses (1 strain), ducks (1 strain), seal (1 strain), and midges (1 strain) (refer to Table 1). Detailed background information for all strains (including GenBank accession numbers, collection time, geographical origin, host type, etc.) is compiled in Supplementary Table S1.

### 3.2. Time-Scaled Phylogenetic Analysis of JEV Based on Complete Genomes

**Figure 2:**
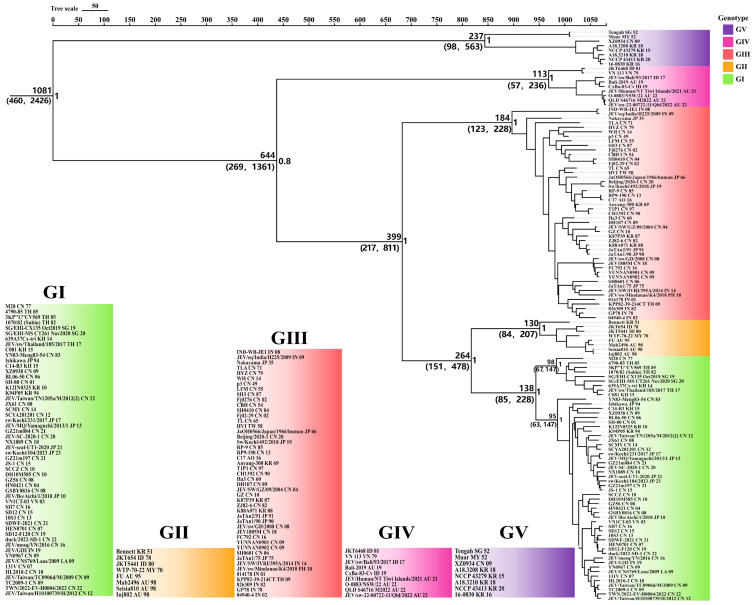
MCC tree of JEV complete genomes. Background colors indicate different genotypes: green for GI, orange for GII, red for GIII, magenta for GIV, and purple for GV. The tMRCA estimates (with 95% HPD values in parentheses) are shown to the left of nodes. Posterior probabilities for branches are indicated to the right of nodes. Branches are labeled following the convention: StrainName_CountryAbbreviation_IsolationYear.

Based on the BF and the 95% HPD interval, the Bayesian skyline model with a relaxed molecular clock was selected as the best-fitting model. Using Bayesian MCMC analysis, an MCC tree was constructed (Figure 2), in which all branch nodes showed posterior probability values greater than 0.8, indicating the robustness of the results. The MCC tree revealed that the time to tMRCA of JEV was estimated to be approximately 1081 years ago (95% HPD, 460–2426), after which it diverged into five genotypes (GI–GV). The tMRCA estimates for each genotype were as follows: GV, 237 years (95% HPD, 98–563); GIII, 184 years (95% HPD, 123–228); GI, 138 years (95% HPD, 85–228); GII, 130 years (95% HPD, 84–207); and GIV, 113 years (95% HPD, 57–236). Emerged sequentially in the evolutionary order of GV, GIII, GI, GII, and GIV. GII appeared contemporaneously with the reemerging GIV JEV detected in Australia in 2022 and GI, which replaced GIII as the predominant lineage across the Asian continent.

To further validate the phylogenetic inference, a ML phylogenetic tree was constructed using IQ-TREE, with the topology largely consistent with the BEAST time-scaled MCC tree (see Supplementary Figure S1), supporting the reliability and robustness of the evolutionary analysis.

### 3.3. Evolutionary Rate and Population Dynamics Analysis of JEV

The mean nucleotide substitution rate of the entire JEV population was estimated to be 3.3 × 10^−4^ substitutions per site per year (S/S/Y) (95% HPD, 1.9 × 10^−4^–4.7 × 10^−4^). The mean substitution rates for each genotype were estimated as follows: GI, 7.3 × 10^−4^ (95% HPD, 4.4 × 10^−4^–1.1 × 10^−3^); GII, 8.2 × 10^−4^ (95% HPD, 5.0 × 10^−4^–1.0 × 10^−3^); GIII, 4.4 × 10^−5^ (95% HPD, 2.3 × 10^−7^–8.9 × 10^−5^); GIV, 1.7 × 10^−3^ (95% HPD, 4.5 × 10^−4^–3.9 × 10^−3^); and GV, 2.2 × 10^−3^ (95% HPD, 4.0 × 10^−8^–8.9 × 10^−3^). The evolutionary rate among the five major genotypes ranked from fastest to slowest as GV > GIV > GII > GI > GIII (Table 2).

**Figure 3:**
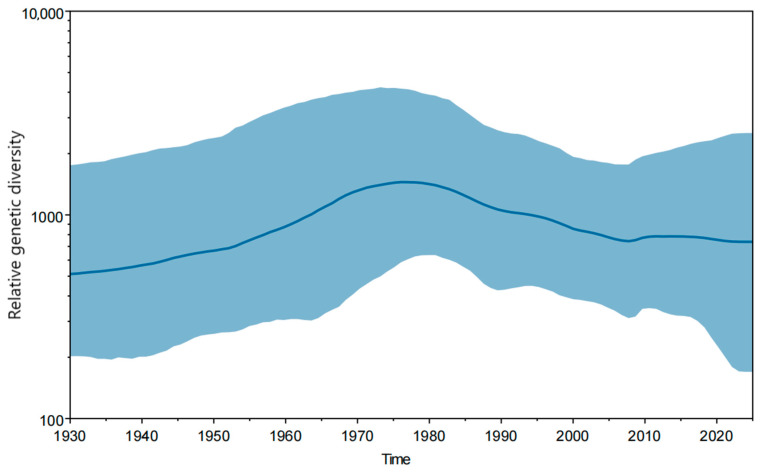
Bayesian skyline plot depicting changes in JEV population diversity over time. The highlighted area represents the 95% HPD interval. The horizontal axis shows time, and the vertical axis shows genetic diversity.

The Bayesian skyline plot depicting the population dynamics of JEV (Figure 3) showed that prior to 1975, the viral population diversity exhibited a steady increase, reaching its peak around 1975, followed by a continuous decline from 1976 to 2010, and subsequently stabilized after 2010.

### 3.4. Structural Similarity Comparison of Genomic Regions Across Five Genotypes of JEV Representative Strains

**Figure 4:**
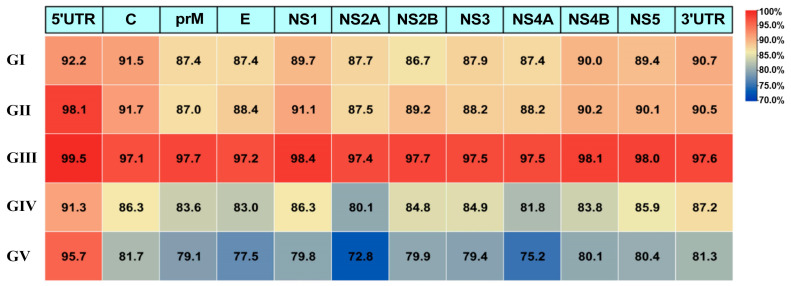
Comparison of genomic structural similarity between representative strains of the five JEV genotypes and the P3 vaccine strain (GIII). The horizontal axis represents the genomic structures of JEV. The vertical axis represents viral strains of different genotypes. Similarity is color-coded from blue (lowest) to red (highest). The percentage identity values (% identity) are calculated with reference to the P3 vaccine strain.

Based on comparative genomic analysis with the P3 vaccine strain, significant differences in sequence similarity were observed among different JEV genotypes (Figure 4). The GIII exhibited extremely high similarity with the P3 strain across all structural regions (97.1–99.5%), whereas other genotypes showed relatively lower similarity, particularly GIV and GV, which displayed the most pronounced divergence. Comparative analysis of key functional regions revealed that sequence similarity between each genotype and the P3 vaccine strain differed most notably in the E, NS2A, and NS4A proteins. As the major antigenic target, the E protein exhibited similarity in the order GIII (97.2%) > GII (88.4%) > GI (87.4%) > GIV (83.0%) > GV (77.5%). The NS2A protein, which plays a critical role in viral assembly and immune evasion, showed similarity in the order GIII (97.4%) > GI (87.7%) > GII (87.5%) > GIV (80.1%) > GV (72.8%). The NS4A protein, involved in the formation of the viral replication complex, exhibited similarity in the order GIII (97.5%) > GII (88.2%) > GI (87.4%) > GIV (81.8%) > GV (75.2%). All three key proteins displayed a gradient pattern of similarity, with GIII showing the highest and GV the lowest, among which NS2A exhibited the greatest degree of variation, with a 24.6 percentage point difference between GIII and GV.

### 3.5. Codon Usage Patterns in the Complete Genomes of Different JEV Genotypes

**Figure 5:**
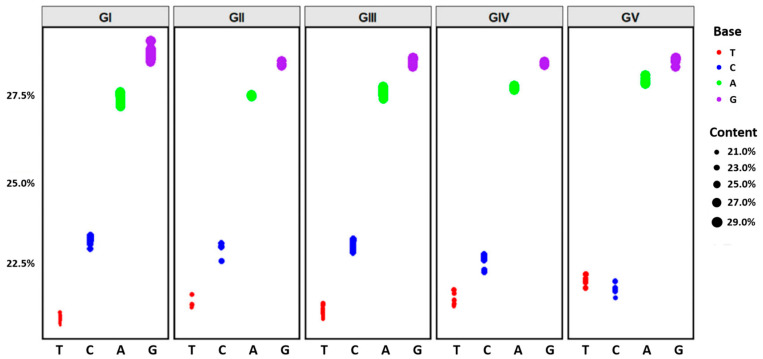
Codon usage patterns in complete genomes of different JEV genotypes. The vertical axis represents nucleotide content. The horizontal axis represents nucleotide types. Colors indicate specific nucleotides: red for T, blue for C, green for A, and purple for G.

Nucleotide composition analysis of JEV revealed similar base composition patterns across the five genotypes (Figure 5). For GI-GIV, the base content followed the order: G > A > C > T. Notably, GV exhibited a distinct pattern with G > A > T > C, where T content exceeded C content. Among the five genotypes, T content ranged from 20.8% to 21.9%, with the lowest value observed in GI (20.8%) and the highest in GV (21.9%). C content ranged from 21.8% to 23.2%, with the lowest value in GV (21.8%) and the highest in GI (23.2%). A content ranged from 27.3% to 27.9%, with the lowest value in GI (27.3%) and the highest in GV (27.9%). G content ranged from 28.4% to 28.7%, with the highest value in GI (28.7%), while GII and GIV both exhibited the lowest value (28.4%). Detailed nucleotide compositions for each genotype are provided in Table 2.

### 3.6. Analysis of Amino Acid Mutation Sites in the E Protein of Recent Human JEV Strains Compared to the Vaccine P3 Strain

**Figure 6:**
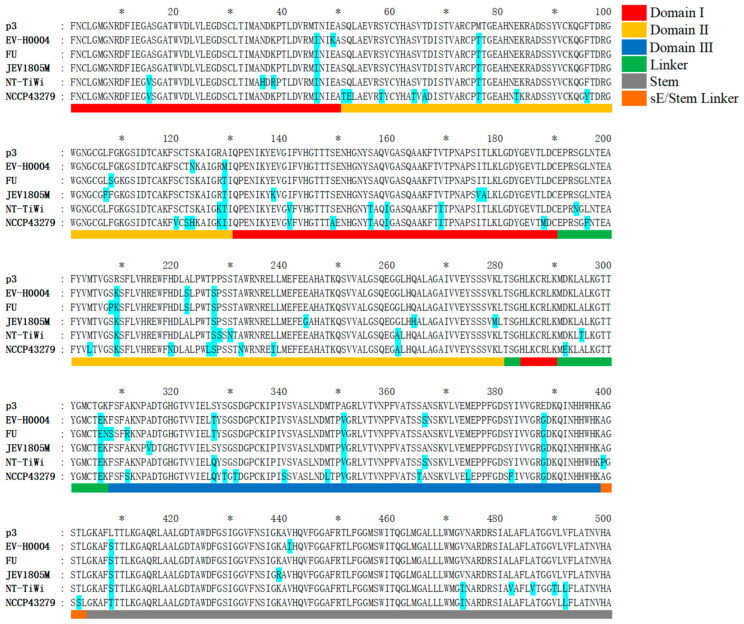
Analysis of amino acid mutation sites in the E protein of latest human JEV strains compared to the vaccine P3 strain. TWN/2022-EV-H0004/2022 (EV-H0004) represents a GI strain; FU represents a GII strain; JEV1805M represents a GIII; JEV/Human/NT_Tiwi Islands/2021 (NT-TiWi) represents a GIV strain; NCCP 43279 represents a GV strain. Amino acids differing from the P3 strain are highlighted in sky blue background. In the scale bar, major ticks are placed every 20 units, with asterisks (*) denoting the intervening minor tick positions. The structural information of the corresponding protein is indicated by the colors below the sequence: red for Domain I, yellow for Domain II, dark blue for Domain III, green for the Linker, gray for the Stem, and orange for the sE/Stem Linker.

The JEV E protein is a typical class II fusion protein, composed of three major domains (DI–DIII) and a C-terminal helical stem–membrane anchor region [37]. Domain I (DI) forms a central β-barrel structure encompassing residues E1–E50, E131–E190, and E284–E290. Domain II (DII) forms a finger-like extension consisting of E51–E130 and E201–E280, which contains the hydrophobic fusion loop required for viral fusion and is a critical region for E protein dimerization and membrane fusion. Domain III (DIII) adopts an immunoglobulin-like globular structure located at the extracellular C-terminus (E308–E398) and often serves as a key target for neutralizing antibodies. A short linker peptide (E291–E307) connects DI and DIII, providing flexibility and acting as a pivot during conformational changes. Following DIII, the helical stem–membrane anchor region (E399–E500) contains two stem helices and a transmembrane helix, serving as essential structural units for viral assembly and membrane fusion (Figure 6).

Compared to the vaccine P3 strain, the GI strain TWN/2022-EV-H0004/2022 exhibited fifteen amino acid variations: two in DI (T46I, E49K); six in DII (M76T, S123N, A129M, R209K, A222S, P227S); and four in DIII (S327T, A351V, A366S, E388G). The GII strain FU displayed sixteen differences: one in DI (T46I); seven in DII (M76T, F108S, A129T, S208P, R209K, A222S, P227S); and five in DIII (F308S, A311R, S327T, A351V, E388G). The GIII JEV1805M showed eighteen differences: four in DI (T46I, E138K, I176V, T177A); eight in DII (M76T, L107F, A129T, R209K, P227S, E244G, Q264H, K279M); and three in DIII (A315V, A351V, E388G). The GIV strain JEV/Human/NT_Tiwi Islands/2021 exhibited thirty differences: eight in DI (A15V, N36H, K38R, T46I, I141V, S156T, V159I, V169I); eight in DII (M76T, R128K, A129T, R209K, P227S, P228S, S230N, G261A); and four in DIII (S327Q, A351V, A366S, E388G). The GV strain NCCP 43279 displayed forty-seven differences: eight in DI (A15V, T46I, I141V, S149A, S156T, V159I, V169I, L188M); twenty-one in DII (S51T, Q52E, S58T, S64T, T66A, M76T, E83T, F96Y, S120V, T122S, S123H, R128K, A129I, M204L, R209K, H219N, T226L, P227S, A232N, L238I, G261A); and eleven in DIII (A311S, S327Q, S329T, S331T, V340S, M348L, A351V, S365T, M374L, Y382F, E388G). Notably, all five genotypes shared nine common mutation sites compared to the P3 strain: T46I, M76T, A129T/M, R209K, P227S, G306E, A351V, E388G, and L408S/T.

### 3.7. Structural and Surface Charge Analysis of the E Protein of Recent Human JEV Strains and the Vaccine P3 Strain

**Figure 7:**
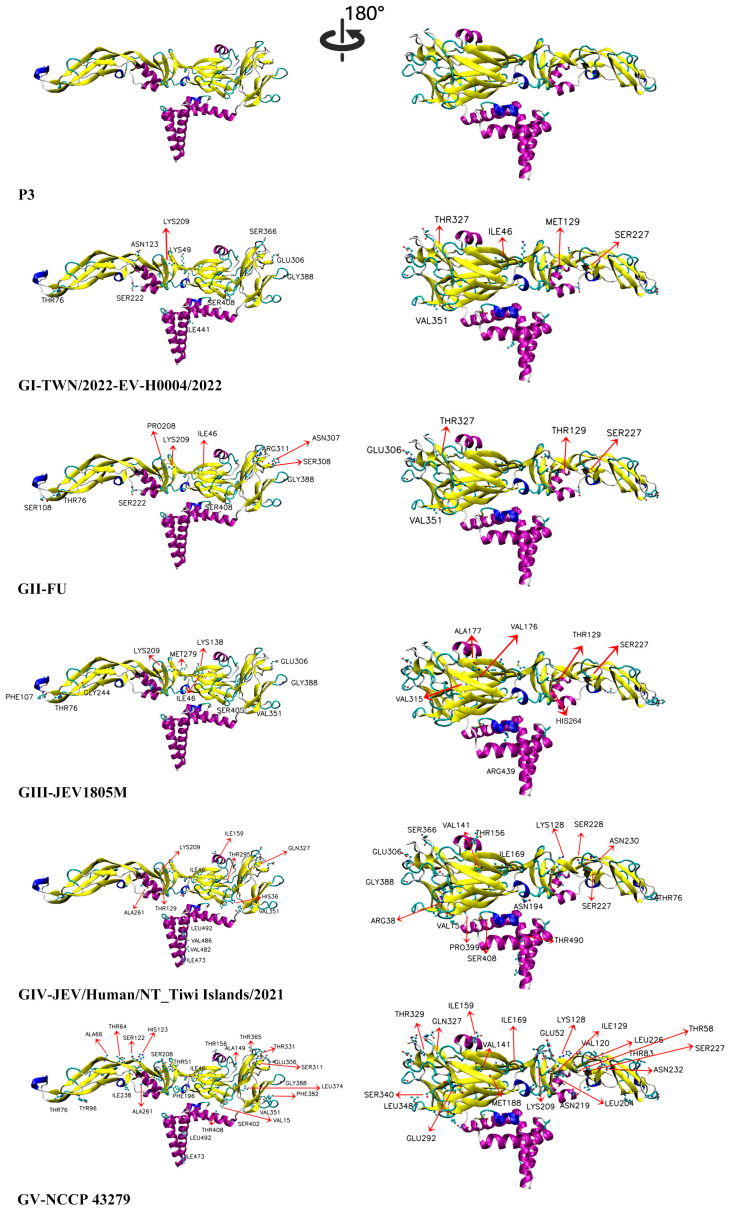
Three-dimensional structural models of the E protein of recent human JEV strains and the vaccine P3 strain. TWN/2022-EV-H0004/2022, FU, JEV1805M, JEV/Human/NT_Tiwi Islands/2021, and NCCP 43279 represent recent human-derived JEV strains (GI–GV). Structural mapping of mutation sites in the E protein. Secondary structure elements are colored as follows: Alpha Helix (purple), 3_10_Helix (blue), Extended_Beta (yellow), Bridge_Beta (light brown), Turn (cyan), Coil (white). Sites differing from the P3 strain are labeled in the structure.

To investigate the potential impact of these mutations on the higher-order structure of the JEV E protein, we conducted a comprehensive assessment through three-dimensional structural modeling and surface electrostatic potential analysis. Figure 7 displays the three-dimensional structural models of representative strains of each genotype colored according to secondary structure elements. Additionally, we provide the P3 vaccine strain structural model in Supplementary Figure S2, which adopts the standard domain coloring scheme (DI: red, DII: yellow, DIII: blue) to facilitate precise domain-level comparison. The results of surface electrostatic potential distribution analysis are presented in Supplementary Figure S3. For the analysis, we defined the criteria for significant physicochemical changes as follows: (1) charge alterations (conversion between positive and negative charges, or between charged and neutral states); (2) hydrophobicity/hydrophilicity changes (Kyte–Doolittle hydropathy index change ≥1); and (3) steric effects (side-chain volume change ≥50 Å^3^). Only residues meeting at least one of these criteria were included in subsequent analyses. It should be emphasized that these analyses are theoretical predictions, and their actual biological functional impacts require experimental validation.

Compared to the P3 strain, the GI strain (TWN/2022-EV-H0004/2022) exhibited 11 mutations with significant physicochemical alterations: eight sites (T46I, M76T, A222S, P227S, G306E, A366S, E388G, L408S) led to notable changes in hydrophobicity/hydrophilicity; amino acid side chains exerted varied steric effects at A129M, G306E, A351V, and E388G; and significant charge changes were observed at E49K, G306E, and E388G. The GII strain (FU) showed 14 mutations with significant physicochemical changes: eleven sites (T46I, M76T, F108S, A129T, A222S, P227S, G306E, F308S, A311R, E388G, L408S) resulted in notable hydrophobicity/hydrophilicity alterations; side chains exhibited differential steric effects at F108S, G306E, F308S, A311R, A351V, and E388G; and significant charge changes occurred at G306E, K307N, A311R, and E388G. The GIII (JEV1805M) displayed 15 mutations with significant physicochemical alterations: ten sites (T46I, M76T, A129T, T177A, P227S, E244G, K279M, G306E, E388G, L408S) led to notable hydrophobicity/hydrophilicity changes; side chains showed varied steric effects at E244G, G306E, A315V, A351V, and E388G; and significant charge changes were observed at E138K, E244G, Q264H, K279M, G306E, and E388G. The GIV strain (JEV/Human/NT_Tiwi Islands/2021) exhibited 18 mutations with significant physicochemical changes: twelve sites (T46I, M76T, A129T, P227S, P228S, G261A, A295T, G306E, A366S, E388G, L408S, V490T) resulted in notable hydrophobicity/hydrophilicity alterations; side chains exerted differential steric effects at A15V, G261A, G306E, S327Q, A351V, E388G, A399P, and A486V; and significant charge changes occurred at N36H, G306E, and E388G. The GV strain (NCCP 43279) showed 22 mutations with significant physicochemical alterations: fifteen sites (T46I, T66A, M76T, E83T, S120V, A129I, S149A, P227S, A232N, G261A, G306E, A311S, V340S, E388G, L408T) led to notable hydrophobicity/hydrophilicity changes; side chains exhibited varied steric effects at A15V, G261A, G306E, S327Q, A351V, E388G, and L408T; and significant charge changes were observed at Q52E, E83T, S123H, H219N, G306E, and E388G. Detailed information on all differential amino acid sites is provided in Supplementary Table S2.

## 4. Discussion

The spatiotemporal evolutionary dynamics of RNA viruses can be effectively elucidated through phylogenetic structural analyses, providing a scientific approach to understanding viral evolution in nature. The time-scaled phylogenetic analysis in this study estimated the tMRCA of JEV to be around the year 1081 (95% HPD, 460–2426). The evolutionary order of the genotypes was determined as GV, GIII, GI, GII, and GIV, with the specific divergence times as follows: GV: 237 years (95% HPD, 98–563); GIII: 184 years (95% HPD, 123–228); GI: 138 years (95% HPD, 85–228); GII: 130 years (95% HPD, 84–207); and GIV: 113 years (95% HPD, 57–236). Previously, a study by Gao et al. reported a TMRCA of JEV over 3255 years ago, with genotypes evolving in the order GV, GIV, GIII, GII, and GI [11]. Our results differ significantly from this earlier finding. This discrepancy is primarily due to the limited dataset used in that study, which included only 2 GV strains, 1 GII strain, and 1 GIV strain, leading to dataset bias. In contrast, the dataset in the present study comprises 126 JEV strains collected between 1935 and 2025, including 9 GIV strains [17,38,39], 8 GV strains [21,40,41], and 8 GII strains [16,42]. The more comprehensive dataset in this study provides more accurate estimates, offering a more reliable reflection of the emergence time and evolutionary order of JEV genotypes.

The substitution rate is an important parameter in understanding virus evolution, as a high mutation rate often leads to strong adaptability and high pathogenicity [43]. The evolutionary rates of JEV vary among the overall population and different genotypes. Our analysis estimated the overall evolutionary rate of JEV at approximately 3.3 × 10^−4^ S/S/Y (95% HPD, 1.9 × 10^−4^–4.7 × 10^−4^). Analysis by genotype revealed significant differences in nucleotide substitution rates, with the ranking from highest to lowest as GV > GIV > GII > GI > GIII, corresponding to 2.2 × 10^−3^, 1.7 × 10^−3^, 8.2 × 10^−4^, 7.3 × 10^−4^, and 4.4 × 10^−5^ S/S/Y, respectively. The observed high evolutionary rate in GV (GII & GIV) merits attention; however, it is important to acknowledge that this estimate is based on limited sequence data currently available. Achieving a more accurate comprehension of its evolutionary dynamics will necessitate the future collection and analysis of additional natural JEV strains. The genotypes GII, GIV, and GV traditionally considered “non-dominant” or geographically restricted were estimated to have higher evolutionary rates than the widely circulating GI and GIII genotypes. A similar pattern has been observed in other RNA viruses. For example, the 2016 Zika virus outbreak in Brazil was caused by a recently diverged, faster-evolving Asian genotype, with an estimated evolutionary rate of 1.0233 × 10^−3^ S/S/Y (95% HPD, 8.2 × 10^−4^–1.2 × 10^−3^) [44]. Furthermore, it is noteworthy that the rapidly evolving GIV genotype, with an estimated evolutionary rate of 1.7 × 10^−3^ S/S/Y (HPD, 4.5 × 10^−4^, 3.9 × 10^−3^), was responsible for a significant JE outbreak in southeastern Australia in 2022, resulting in 45 human cases and 7 fatalities [10,45]. Additionally, the GV genotype, which re-emerged in mosquito samples from Tibet, China (78°25° E–99°06° E, 26°50° N–36°53° N), in 2009, has, within just 16 years, spread from its initial location to South Korea (125.1° E–129.3° E, 34.3° N–38.6° N), where it has rapidly become the dominant circulating strain and has caused multiple cases of JE. Our team’s previous experimental findings indicate that conventional vaccines developed against the GIII genotype provide limited immune protection against the GV genotype [46]. Collectively, these findings suggest that an increase in evolutionary rate may be associated with the acquisition of new transmission advantages or adaptation to novel hosts and environments.

The genetic diversity center of JEV is located in the Indonesia–Malaysia region (10° S to 10° N), which is considered to be the origin region of the JEV [8]. Interestingly, different genotypes exhibit distinct latitudinal distribution preferences during their spread. Specifically, GI, GIII, and GV have successfully adapted to and dominated circulation in the mid-latitudes of the Northern Hemisphere (20°–45° N), whereas GII and GIV have become the predominant genotypes in the Southern Hemisphere (Australia, 10°–45° S). This contrasting North–South distribution pattern may reflect co-evolution and ecological adaptation of the virus with local vectors, hosts, and climatic conditions. Notably, JEV GII has previously caused JE outbreaks on Australian islands [42], and in 2022, GIV re-emerged in southeastern Australia, causing a sudden outbreak [10]. Our analysis of viral population evolutionary rates indicates that both GII and GIV JEV evolve rapidly. Moreover, the virus can spread over long distances via migratory birds along the Australia–East Asia flyway or through mosquito dispersal assisted by monsoon winds. These factors collectively increase the risk of JEV spreading into new regions [47]. Active surveillance of JEV in pigs, birds, and mosquito vectors in non-traditional endemic areas including Europe, Africa, and the Americas can facilitate early detection of viral introductions.

There are no specific antiviral drugs available for JEV, and its prevention and control rely primarily on vaccination. The E protein, the major surface glycoprotein of JEV, performs multiple biological functions during infection. Its receptor-binding domain specifically recognizes host cell surface molecules such as glycosaminoglycans, mediating viral attachment and entry into target cells [48]. Structural biology analyses in this study revealed significant differences in key antigenic structures of the E protein between current circulating human isolates and vaccine strains, particularly in the capsid protein, the fusion loop of DII, and the receptor-binding and neutralizing epitope regions of DIII. These accumulated mutations may reduce antibody binding efficiency, thereby weakening the cross-protective effect elicited by vaccination. These molecular mechanisms provide a plausible explanation for recent epidemiological changes. In outbreaks in Australia (GIV) and South Korea (GV), most infections occurred in adults, deviating from the traditional pattern of JEV primarily affecting children [45,49]. This suggests that re-emerging non-dominant genotypes may have undergone antigenic changes and could potentially exhibit altered pathogenic phenotypes, including neurotropism. It is crucial to acknowledge that the suggested association between E protein mutations and reduced vaccine efficacy is predominantly based on in silico analyses. Consequently, this hypothesis requires further validation through neutralization assays and other experimental methodologies. To thoroughly comprehend the phenotypic implications of these mutations, systematic investigations into the pathobiology of various JEV genotypes are urgently necessary. With the continuous development and refinement of advanced research models such as brain organoids, future investigations can leverage these in vitro systems to reveal phenotypic differences and underlying mechanisms of neural invasiveness and host responses among distinct viral genotypes [50].

## 5. Conclusions

The estimated time to tMRCA of JEV is 1081 (95% HPD, 460–2426), with the five genotypes evolving in the order of GV, GIII, GI, GII, and GIV. Notably, the currently non-dominant genotypes (GII, GIV, and GV) not only exhibit faster evolutionary rates but also differ in the characteristics of their infected populations compared with the previously dominant genotypes (GIII and GI), which primarily affected children. As there are still no specific antiviral drugs available for JEV, vaccination remains the main preventive measure. Furthermore, recent research indicates that current vaccines derived from JEV GIII confer low protection against the emerging JEV GV [46,51], which may limit their ability to effectively induce cross-protective immunity against these non-dominant genotypes. The limitations of vaccine-induced protection, together with the absence of specific therapeutic options, collectively exacerbate the public health threat posed by these emerging non-dominant genotypes. Given their enhanced host adaptability and cross-species transmission potential, along with the increasing frequency of global climate change and international mobility, the risk of JEV spreading to new regions such as Europe, Africa, and the Americas continues to rise. Therefore, establishing a globally integrated surveillance network encompassing humans, animals, and vectors, and accelerating the development of next-generation vaccines that include emerging genotypes, have become urgent priorities for global public health.

## Figures and Tables

**Table 1:** Distribution, genotype, vector, host, and isolation data of JEV strains utilized in the current study.

Continent	Country	Genotype					Vector/Host								Year									
		I	II	III	IV	V	Mosquito	Midges	Human	Pig	Horse	Duck	Cattle	Seal	1930s	1940s	1950s	1960s	1970s	1980s	1990s	2000s	2010s	2020s
Asia	China	34	/	28	/	1	33	1	15	14	/	1	/	/	/	1	2	3	3	5	2	21	19	7
	Cambodia	3	/	/	/	/	1	/	1	1	/	/	/	/	/	/	/	/	/	/	/	/	3	/
	India	1	/	7	/	/	2	/	4	1	1	/	/	/	/	/	/	/	1	1	/	4	2	/
	Indonesia	/	2	/	3	/	4	/	/	1	/	/	/	/	/	/	/	/	1	2	/	/	2	/
	Japan	6	/	6	/	/	2	/	2	6	/	/	1	1	1	/	/	1	1	/	3	4	/	2
	Korea	1	/	/	/	/	1	/	/	/	/	/	/	/	/	/	/	/	/	/	1	/	/	/
	Laos	1	/	/	/	/	/	/	1	/	/	/	/	/	/	/	/	/	/	/	/	1	/	/
	Malaysia	/	1	/	/	1	1	/	1	/	/	/	/	/	/	/	1	/	1	/	/	/	/	/
	Philippines	/	/	1	/	/	/	/	/	1	/	/	/	/	/	/	/	/	/	/	/	/	1	/
	Singapore	2	/	/	/	1	2	/	1	/	/	/	/	/	/	/	1	/	/	/	/	/	1	1
	South Korea	1	1	3	/	5	2	/	6	2	/	/	/	/	/	/	1	1	/	2	/	/	5	1
	Thailand	4	/	1	/	/	2	/	2	1	/	/	/	/	/	/	/	/	/	3	/	1	1	/
	Viet Nam	1	/	/	1	/	1	/	1	/	/	/	/	/	/	/	/	/	1	/	/	1	/	/
Africa	Angola	/	/	1	/	/	/	/	1	/	/	/	/	/	/	/	/	/	/	/	/	/	1	/
Oceania	Australia	/	4	/	5	/	1	/	3	5	/	/	/	/	/	/	/	/	/	/	4	/	1	4

Note: “/” indicates that the data are not available.

**Table 2:** The nucleotide content and Bayesian Markov Chain Monte Carlo analysis for JEV.

JEV Genotypes	tMRCA (95%HPD)	Substitution Rate S/S/Y (95%HPD)	Nucleotide Content Percentage
JEV all five genotypes	1081 (460, 2426)	3.3 × 10^−4^ (1.9 × 10^−4^, 4.7 × 10^−4^)	T: 21.0 C: 23.0 A: 27.5 G: 28.5
GI	138 (85, 228)	7.3 × 10^−4^ (4.4 × 10^−4^, 1.1 × 10^−3^)	T: 20.8 C: 23.2 A: 27.3 G: 28.7
GII	130 (84, 207)	8.2 × 10^−4^ (5.0 × 10^−4^, 1.0 × 10^−3^)	T: 21.3 C: 22.9 A: 27.4 G: 28.4
GIII	184 (123, 228)	4.4 × 10^−5^ (2.3 × 10^−7^, 8.9 × 10^−5^)	T: 21.0 C: 23.0 A: 27.5 G: 28.5
GIV	113 (57, 236)	1.7 × 10^−3^ (4.5 × 10^−4^, 3.9 × 10^−3^)	T: 21.4 C: 22.6 A: 27.6 G: 28.4
GV	237 (98, 563)	2.2 × 10^−3^ (4.0 × 10^−8^, 8.9 × 10^−3^)	T: 21.9 C: 21.8 A: 27.9 G: 28.5

## Data Availability

The original contributions presented in the study are included in the article and Supplementary Materials, further inquiries can be directed to the corresponding authors.

## Supplementary Materials

The following supporting information can be downloaded at: https://www.mdpi.com/article/10.3390/microorganisms13122792/s1, Data S1: The JEV sequences used in this study; Figure S1: Maximum likelihood tree of JEV complete genomes. Triangles represent strains belonging to the same genotype. Posterior probability values for each cluster are displayed to the left of the nodes; Figure S2: Three-dimensional structure of the P3 E protein with domains colored. The structure is color-coded as follows: Domain I (red), Domain II (yellow), Domain III (blue), Linker (green), Stem (gray), and sE/Stem Linker (orange); Figure S3: Electrostatic potential models of the E protein of recent human JEV strains and the vaccine P3 strain. Surface charge distribution of the E protein. Electrostatic potentials are color-coded: blue represents positive charge, red represents negative charge, and white indicates neutral regions; Table S1: The JEV isolates analyzed in the current study; Table S2: A comparative analysis of mutation sites within the three-dimensional structure of the E protein between recent human JEV strains and the P3 strain.
