# Supplementary material for: Non-Dominant Genotypes (GII, GIV and GV) of Japanese Encephalitis Virus Exhibit an Elevated Evolutionary Rate in Nature
Source: Microorganisms. 2025 Dec 8;13(12):2792. doi: 10.3390/microorganisms13122792 (PMC12735678; doi:10.3390/microorganisms13122792)
Supplement: Supplementary file 1 [file microorganisms-13-02792-s001.zip › Figure S1: Maximum likelihood tree of JEV complete genomes.pdf]

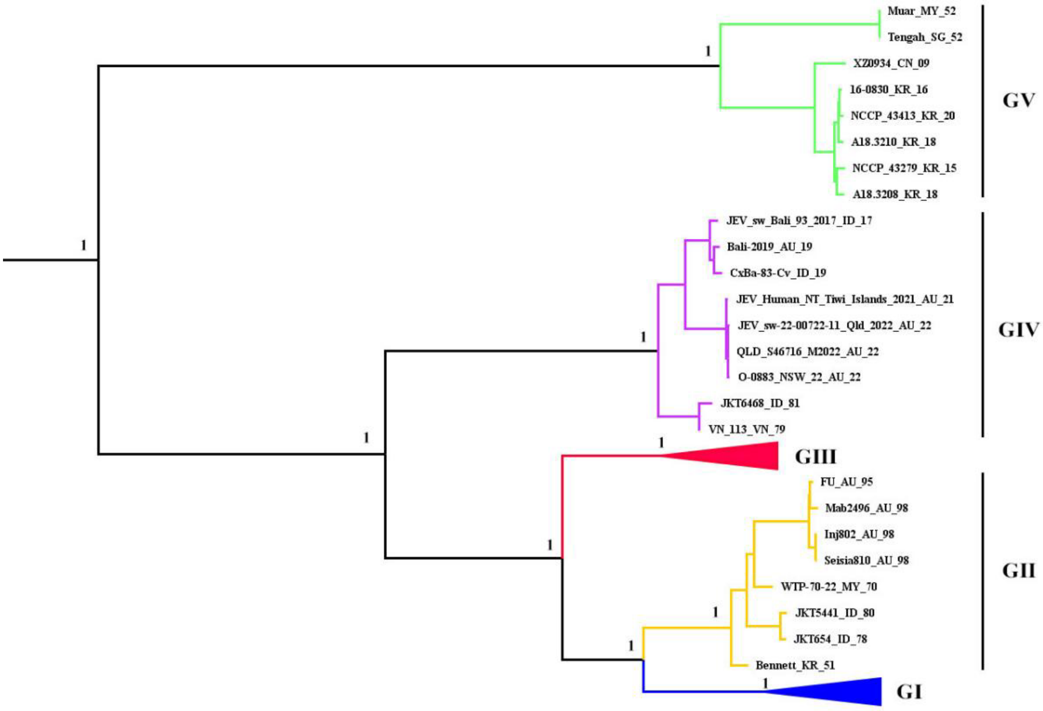

Figure S1. Maximum likelihood tree of JEV complete genomes. Triangles represent strains belonging to the same genotype. Posterior probability values for each cluster are displayed to the left of the nodes.
