# Supplementary material for: Non-Dominant Genotypes (GII, GIV and GV) of Japanese Encephalitis Virus Exhibit an Elevated Evolutionary Rate in Nature
Source: Microorganisms. 2025 Dec 8;13(12):2792. doi: 10.3390/microorganisms13122792 (PMC12735678; doi:10.3390/microorganisms13122792)
Supplement: Supplementary file 1 [file microorganisms-13-02792-s001.zip › Figure S2: Three-dimensional structure of the P3 E protein with domains colored.pdf]

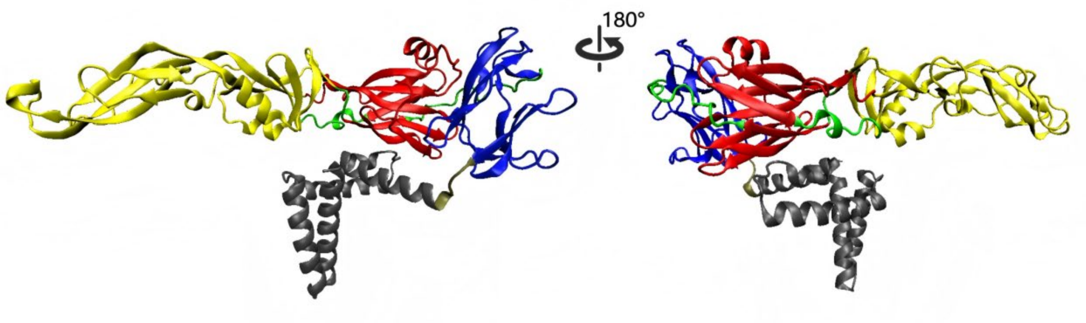

Figure S2. Three-dimensional structure of the P3 E protein with domains colored. The structure is color-coded as follows: Domain I (red), Domain II (yellow), Domain III (blue), Linker (green), Stem (gray), and sE/Stem Linker (orange).
