# Supplementary material for: Non-Dominant Genotypes (GII, GIV and GV) of Japanese Encephalitis Virus Exhibit an Elevated Evolutionary Rate in Nature
Source: Microorganisms. 2025 Dec 8;13(12):2792. doi: 10.3390/microorganisms13122792 (PMC12735678; doi:10.3390/microorganisms13122792)
Supplement: Supplementary file 1 [file microorganisms-13-02792-s001.zip › Figure S3:Electrostatic potential models of the E protein of recent human JEV strains and the vaccine P3 strain.pdf]

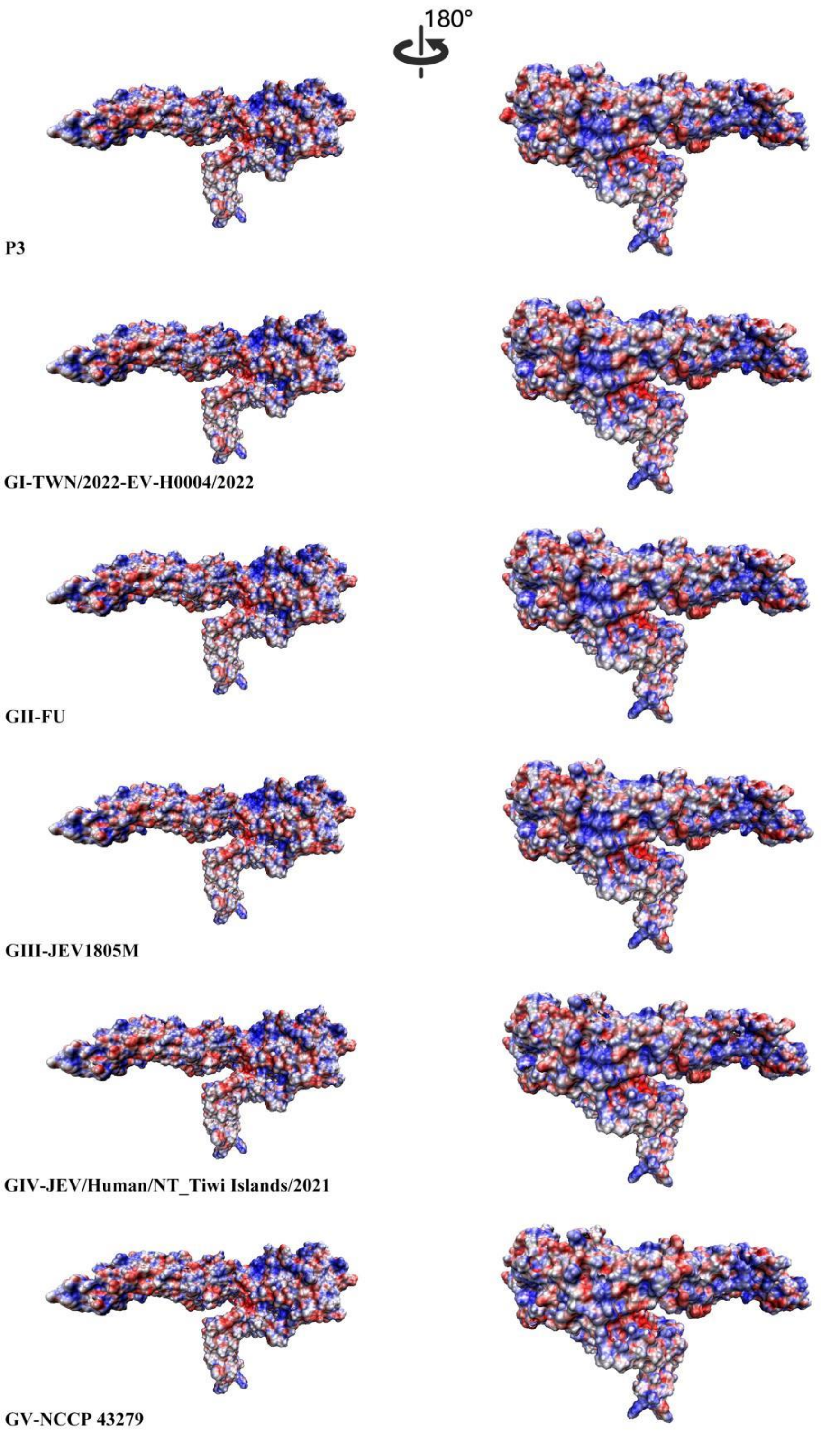

Figure S3. Electrostatic potential models of the E protein of recent human JEV strains and the vaccine P3 strain. Surface charge distribution of the E protein. Electrostatic potentials are color-coded: blue represents positive charge, red represents negative charge, and white indicates neutral regions.
