# Supplementary material for: Non-Dominant Genotypes (GII, GIV and GV) of Japanese Encephalitis Virus Exhibit an Elevated Evolutionary Rate in Nature
Source: Microorganisms. 2025 Dec 8;13(12):2792. doi: 10.3390/microorganisms13122792 (PMC12735678; doi:10.3390/microorganisms13122792)
Supplement: Supplementary file 1 [file microorganisms-13-02792-s001.zip › Table S1:The JEV isolates analyzed in the current study.pdf]

Table S1. The JEV isolates analyzed in the current study.

NO	Strains	Host	Date	Locations	Genotype	Locus
1	M28	Culex pseudovishnui	1977	China	I	JF706279
2	1070/82 (Subin)	Homo sapiens	1982	Thailand	I	GQ902059
3	YN83-Meng83-54	Lasiohelea taiwana Shiraki	1983	China	I	JF706282
4	3KP"U"CV569	mosquito	1985	Thailand	I	GQ902060
5	4790-85	Homo sapiens	1985	Thailand	I	GQ902062
6	Ishikawa	Culex tritaeniorhynchus	1994	Japan	I	AB051292
7	K94P05	mosquito	1994	Korea	I	AF045551
8	SH-80	Culex tritaeniorhynchus	2001	China	I	JN381848
9	VN1CT-03	Culex tritaeniorhynchus	2003	Viet Nam	I	PP336429
10	HN0421	Culex	2004	China	I	JN381841
11	BL06-50	Culex tritaeniorhynchus	2006	China	I	JF706270
12	131V	Homo sapiens	2007	China	I	GU205163
13	HEN0701	Swine	2007	China	I	FJ495189
14	JX61	pig	2008	China	I	GU556217
15	GSBY0816	Culex tritaeniorhynchus	2008	China	I	JN381842
16	GZ56	Homo sapiens	2008	China	I	HM366552
17	JEV/CNS769/Laos/2009	Homo sapiens	2009	Laos	I	KC196115
18	YN0967	Culex tritaeniorhynchus	2009	China	I	JF706268
19	JEV/Taiwan/TC0906d/M/2009	Culex tritaeniorhynchus	2009	China	I	KF667320
20	XZ0938	mosquito	2009	China	I	HQ652538
21	TC2009-1	mosquito	2009	China	I	JF499790
22	SCCZ	mosquito	2010	China	I	KU351667
23	HL2010-2	mosquito	2010	China	I	JQ031753
24	JEV/Bo/Aichi/1/2010	Bos taurus	2010	Japan	I	AB853904
25	DH10M585	Culex tritaeniorhynchus mosquitoes	2010	China	I	KT957421
26	K12IN0325	Wild boar	2010	South Korea	I	OR605600
27	SCYA201201	Sus scrofa	2012	China	I	KM658163
28	JEV/Taiwan/TN1205a/M/2012(2)	Culex tritaeniorhynchus	2012	China	I	KF667325
29	JEV/Taiwan/H10100739/H/2012	Homo sapiens	2012	China	I	KF667324

Continued Table S1. The JEV isolates analyzed in the current study.

NO	Strains	Host	Date	Locations	Genotype	Locus
30	JEV/MQ/Yamaguchi/2013/1	Culex tritaeniorhynchus	2013	Japan	I	AB981183
31	10S3	pig	2013	China	I	MF542268
32	SCMY	swine	2014	China	I	KU351668
33	639A37Cx-tri	Culex tritaeniorhynchus	2014	Cambodia	I	KY927815
34	JS-1	Culex tritaeniorhynchus	2015	China	I	KX357114
35	C081	Homo sapiens	2015	Cambodia	I	KY927816
36	C14-B3	pig	2015	Cambodia	I	KY927817
37	SD12	swine	2015	China	I	MH753127
38	JEV/mosq/YN/2016	Mosquito	2016	China	I	MH385014
39	SH7	Culex tritaeniorhynchus	2016	China	I	MH753129
40	JEV/sw/Thailand/185/2017	Sus scrofa	2017	Thailand	I	LC461958
41	sw/Kochi/231/2017	Sus scrofa	2017	Japan	I	LC708275
42	NX1889	Homo sapiens	2018	China	I	MT134112
43	SD12-F120	pig	2019	China	I	MN544779
44	SG/EHI-CX135_Oct2019	Culex	2019	Singapore	I	ON804798
45	JEV/GIII	Culex tritaeniorhynchus	2019	India	I	ON875960
46	JEV-SC-2020-1	Sus scrofa	2020	China	I	OK423757
47	SG/EHI-MS_CT261_Nov2020	Culex tritaeniorhynchus	2020	Singapore	I	ON804799
48	GZ21m084	Armigeres subalbatus	2021	China	I	PV026205
49	GZ21m197	Culex	2021	China	I	PV026211
50	SDWF-2021	Culex	2021	China	I	OR531689
51	JEV-seal-UT1-2020	Phoca vitulina	2021	Japan	I	LC687612
52	TWN/2022-EV-H0004/2022	Homo sapiens	2022	China	I	PQ584053
53	duck/2022-SD-1	duck	2022	China	I	OR711406
54	sw/Kochi/104/2023	Sus scrofa	2023	Japan	I	LC786330
55	FU	Human blood	1995	Australia	II	AF217620
56	JKT5441	mosquito	1980	Indonesia	II	OR119827
57	WTP-70-22	mosquito	1970	Malaysia	II	HQ223286
58	Bennett	Homo sapiens	1951	South Korea	II	HQ223285
59	JKT654	mosquito	1978	Indonesia	II	HQ223287
60	Inj802	porcine	1998	Australia	II	MT253735
61	Seisia810	porcine	1998	Australia	II	MT253736

Continued Table S1. The JEV isolates analyzed in the current study.

NO	Strains	Host	Date	Locations	Genotype	Locus
62	Mab2496	porcine	1998	Australia	II	MT253737
63	Nakayama	Human brain	1935	Japan	III	EF571853
64	p3	mosquito	1949	China	III	U47032
65	CBH	CSF	1954	China	III	JN381860
66	LFM	Human blood	1955	China	III	JN381863
67	Ha3	CSF	1960	China	III	JN381872
68	HVI	Mosquito	1965	China	III	AF098735
69	TL	human	1965	China	III	AF098737
70	JaOH0566/Japan/1966/human	human	1966	Japan	III	AY508813
71	Anyang-300	pig	1969	South Korea	III	KT447437
72	TLA	CSF	1971	China	III	JN381868
73	JaTAn1/75	Sus scrofa	1975	Japan	III	AB551990
74	GP78	Human	1978	India	III	AF075723
75	HYZ	Patient blood	1979	China	III	JN381853
76	826309	Homo sapiens	1982	India	III	PP336430
77	ZJ82-6	Culex tritaeniorhynchus	1982	China	III	KY650724
78	RP-9	mosquito	1985	China	III	AF014161
79	SH3	CSF	1987	China	III	JN381864
80	K87P39	mosquito	1987	South Korea	III	AY585242
81	K88A071	Culex tritaeniorhynchus	1988	South Korea	III	KR908703
82	DH107	Aedes lineatopennis	1989	China	III	JN381873
83	CH1392	Culex tritaeniorhynchus	1990	China	III	AF254452
84	JaTAn1/90	Sus scrofa	1990	Japan	III	AB551991
85	JaTAn2/91	Sus scrofa	1991	Japan	III	AB551992
86	T1P1	Armigeres subalbatus	1997	China	III	AF254453
87	014178	Human blood	2001	India	III	EF623987
88	Fj02-29	CSF	2002	China	III	JF706273
89	04940-4	Culex quinquefasciatus	2002	India	III	EF623989
90	Fj0276	Human blood	2002	China	III	JN381867
91	SH0410	Culex tritaeniorhynchus	2004	China	III	JN381856
92	JEV/SW/GZ/09/2004	pig	2004	China	III	KF297916
93	SH0601	mosquito	2006	China	III	EF543861
94	IND-WB-JE1	Homo sapiens	2008	India	III	JX050179

Continued Table S1. The JEV isolates analyzed in the current study.

NO	Strains	Host	Date	Locations	Genotype	Locus
95	JEV/sw/GD/2008	pig	2008	China	III	KX965684
96	KPP82-39-214CT	mosquito	2009	Thailand	III	GQ902063
97	JEV/eq/India/H225/2009	horse	2009	India	III	JX131374
98	YUNNAN0902	Sus scrofa	2009	China	III	JQ086763
99	YUNNAN0901	mosquito	2009	China	III	JQ086762
100	GZ	swine	2010	China	III	KC915016
101	RP9-190	Culex tritaeniorhynchus	2013	China	III	KF907505
102	JEV/SW/IVRI/395A/2014	Swine	2014	India	III	KP164498
103	WH	pig	2014	China	III	MZ923735
104	C17	Homo sapiens	2016	Angola	III	KX945367
105	FC792	swine	2016	China	III	MF002373
106	JEV1805M	Homo sapiens	2018	China	III	MN639770
107	JEV/sw/Mindanao/K4/2018	Sus scrofa	2018	Philippines	III	LC461960
108	Sw/Kochi/492/2018	Sus scrofa	2019	Japan	III	LC704641
109	Beijing/2020-1	mosquito	2020	China	III	OP588746
110	VN 113	Homo sapiens	1979	Viet Nam	IV	KU705228
111	JKT6468	mosquito	1981	Indonesia	IV	AY184212
112	JEV/sw/Bali/93/2017	Sus scrofa	2017	Indonesia	IV	LC461961
113	Bali 2019	Homo sapiens	2019	Australia	IV	MT253731
114	19CxBa-83-Cv	Culex vishnui	2019	Indonesia	IV	LC579814
115	JEV/Human/NT_Tiwi Islands/2021	Homo sapiens	2021	Australia	IV	OM867669
116	JEV/sw-22-00722-11/Qld/2022	swine	2022	Australia	IV	ON624132
117	QLD_S46716_M2022	mosquito	2022	Australia	IV	OR965960
118	O-0883/NSW/22	swine	2022	Australia	IV	OP904182
119	Muar	Homo sapiens	1952	Malaysia	V	HM596272
120	Tengah	Homo sapiens	1952	Singapore	V	KM677246
121	XZ0934	mosquito	2009	China	V	JF915894
122	NCCP 43279	Homo sapiens	2015	South Korea	V	PP478074
123	16-0830	Culex orientalis	2016	South Korea	V	MT568540
124	A18.3210	Culex bitaeniorhynchus	2018	South Korea	V	MT568538
125	A18.3208	Culex bitaeniorhynchus	2018	South Korea	V	MT568539
126	NCCP 43413	mosquito	2020	South Korea	V	OR500440

Note: The table comprises a total of 126 complete JEV genome sequences, covering five genotypes: GI (54 strains), GII (8 strains), GIII (47 strains), GIV (9 strains), and GV (8 strains). The strains were primarily derived from mosquitoes (55 strains), humans (34 strains),

and pigs (32 strains), with additional sources including cattle (1 strain), horses (1 strain), ducks (1 strain), seal (1 strain), and midges (1 strain). The temporal range of these strains spans from 1935 to 2023.
