# Supplementary material for: Non-Dominant Genotypes (GII, GIV and GV) of Japanese Encephalitis Virus Exhibit an Elevated Evolutionary Rate in Nature
Source: Microorganisms. 2025 Dec 8;13(12):2792. doi: 10.3390/microorganisms13122792 (PMC12735678; doi:10.3390/microorganisms13122792)
Supplement: Supplementary file 1 [file microorganisms-13-02792-s001.zip › Table S2:A comparative analysis of mutation sites within the three-dimensional structure of the E protein between recent human JEV strains and the P3 strain.pdf]

Table S2. A comparative analysis of mutation sites within the three-dimensional structure of the E protein between recent human JEV strains and the P3 strain.

Genotype	Strain	Mutation sites	Standard amino acid	Mutated amino acid	Structure
GI	EV-H0004	46	T	I	DI
		49	E	K	
		76	M	T	DII
		123	S	N	
		129	A	M	
		209	R	K	
		222	A	S	
		227	P	S	
		306	G	E	Linker
		327	S	T	DIII
		351	A	V	
		366	A	S	
		388	E	G	
		408	L	S	Stem
		441	V	I	
GII	FU	46	T	I	DI
		76	M	T	DII
		108	F	S	
		129	A	T	
		208	S	P	
		209	R	K	
		222	A	S	
		227	P	S	
		306	G	E	Linker
		307	K	N	
		308	F	S	DIII
		311	A	R	
		327	S	T	
		351	A	V	
		388	E	G	
		408	L	S	Stem
GIII	JEV1805M	46	T	I	DI
		76	M	T	DII
		107	L	F	
		129	A	T	

Continued Table S2. A comparative analysis of mutation sites within the three-dimensional structure of the E protein between recent human JEV strains and the P3 strain.

Genotype		Strain	Mutation sites	Standard amino acid	Mutated amino acid
		138	E	K	
		176	I	V	DI
		177	T	A	
		209	R	K	
		227	P	S	
		244	E	G	DII
GIII	JEV1805M	264	Q	H	
		279	K	M	
		306	G	E	Linker
		315	A	V	
		351	A	V	DIII
		388	E	G	
		408	L	S	Stem
		439	K	R	Stem
		15	A	V	
		36	N	H	
		38	K	R	DI
		46	T	I	
		76	M	T	
		128	R	K	DII
		129	A	T	
		141	I	V	
		156	S	T	DI
		159	V	I	
GIV	NT-TiWi	169	V	I	
		194	S	N	Linker
		209	R	K	
		227	P	S	
		228	P	S	DII
		230	S	V	
		261	G	A	
		295	A	T	Linker
		306	G	E	
		327	S	Q	
		351	A	V	DIII
		366	A	S	

Continued Table S2. A comparative analysis of mutation sites within the three-dimensional structure of the E protein between recent human JEV strains and the P3 strain.

Genotype	Strain	Mutation sites	Standard amino acid	Mutated amino acid	Genotype
GIV	NT-TiWi	388	E	G	DIII
		399	A	P	sE/Stem Linker
		408	L	S	Stem
		473	V	I	
		482	L	V	
		486	A	V	
		490	V	T	
		492	V	L	
GV	NCCP43279	15	A	V	DI
		46	T	I	
		51	S	T	DII
		52	Q	E	
		58	S	T	
		64	S	T	
		66	T	A	
		76	M	T	
		83	E	T	
		96	F	Y	
		120	S	V	
		122	T	S	
		123	S	H	
		128	R	K	
		129	A	I	
		141	I	V	DI
		149	S	A	
		156	S	T	
		159	V	I	
		169	V	I	
		188	L	M	
		196	L	F	Link
		204	M	L	DII
		208	S	P	
		209	R	K	
		219	H	N	
		226	T	L	
		227	P	S	

Continued Table S2. A comparative analysis of mutation sites within the three-dimensional structure of the E protein between recent human JEV strains and the P3 strain.

Genotype	Strain	Mutation sites	Standard amino acid	Mutated amino acid	Genotype
GV	NCCP43279	232	A	N	DII
		238	L	I	
		261	G	A	
		292	D	E	Linker
		306	G	E	
		311	A	S	DIII
		327	S	Q	
		329	S	T	
		331	S	T	
		340	V	S	
		348	M	L	
		351	A	V	
		365	S	T	
		374	M	L	
		382	Y	F	
		388	E	G	
		402	T	S	sE/Stem Linker
		408	L	T	Stem
		473	V	I	
		492	V	L	

Notes: TWN/2022-EV-H0004/2022 (EV-H0004) represents a GI strain; FU represents a GII strain; JEV1805M represents a GIII strain; JEV/Human/NT_Tiwi Islands/2021 (NT-TiWi) represents a GIV strain; NCCP 43279 represents a GV strain. The structure is color-coded as follows: Domain I (red), Domain II (yellow), Domain III (blue), Linker (green), Stem (gray), and sE/Stem Linker (orange).
